# Tuning Ionic Liquids with Charged Polyhedral Oligomeric Silsesquioxane Nanoparticles for Highly Conductive Quasi-Solid Electrolytes

**DOI:** 10.1021/acs.nanolett.5c02053

**Published:** 2025-06-06

**Authors:** Soorya Koymeth, Marian Paluch, Mateusz Dulski, Zaneta Wojnarowska

**Affiliations:** † Institute of Physics, 49568 University of Silesia in Katowice, 75 Pułku Piechoty 1A, 41−500 Chorzów, Poland; ‡ Institute of Material Science, University of Silesia in Katowice, 75 Pułku Piechoty 1A, 41−500 Chorzów, Poland

**Keywords:** ionic liquids, quasi-solid electrolytes, dielectric spectroscopy, thixotropy, nanoparticles

## Abstract

Electrolytes are fundamental materials that have been used in various electrochemical devices, including fuel cells and batteries. Herein, we report a new class of quasi-solid electrolytes based on ionic liquids (ILs) and multiply charged polyhedral oligomeric silsesquioxane (POSS) nanoparticles that overcome the traditional conductivity–mechanical stability trade-off in solid electrolytes. By precisely controlling the stoichiometric interaction between octa-charged POSS nanoparticles and selected ILs, we achieve unique combinations of properties: room-temperature ionic conductivity σ_dc_^RT^ up to 4 mS/cm, matching or exceeding the parent ILs; reversible shear-thinning behavior enabling easy processing; and exceptional long-term stability against phase separation. Systematic characterization reveals that the 30 wt % POSS loading enhances interfacial charge transfer near the NPs or creates an optimal percolating network where cation–nanoparticle interactions favor fast anion transport. At the same time, the charged POSS framework provides mechanical stability to the quasi-solid electrolyte.

## Graphical abstract

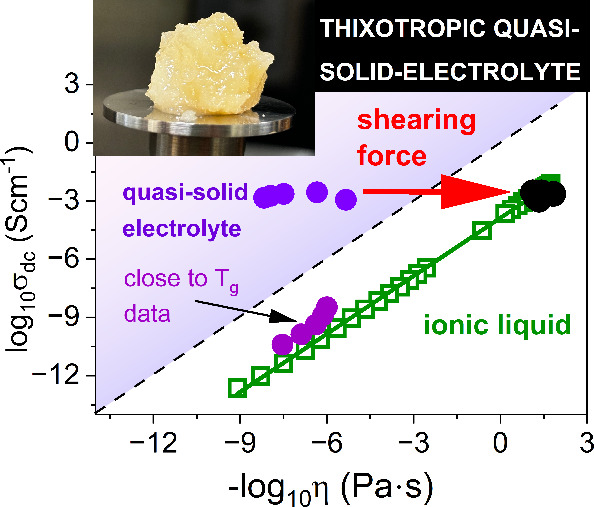

The efficient production, storage, and use of energy have emerged as major contemporary concerns. As a result, there is an increasing need for low-emission cars, clean and dependable energy sources for businesses and homes, and contemporary electrochemical devices made of eco-friendly materials that can produce energy at a reasonable price. Therefore, designing effective electrolytes for a variety of electrochemical applications has been the most often investigated area over the past 10 years.^1–3^

Due to their unique combination of properties such as an exceptionally wide electrochemical window (enabling stability against both reduction and oxidation), high ionic conductivity (reaching 27 mS/cm at room temperature), negligible vapor pressure, nonflammability, and thermal stability, ionic liquids (ILs) have emerged as promising electrolyte candidates.^4,5^ These molten salts, composed entirely of ions, offer additional advantages through their tunable physicochemical properties: characteristics such as density, thermal behavior, and conductivity can be precisely adjusted by selecting appropriate cation–anion combinations. However, their liquid state and relatively low viscosity pose practical challenges, particularly, the risk of leakage in electrochemical devices. This limitation has driven significant research interest in solid-state electrolytes (SSEs), which combine ionic transport functionality with separator capabilities while addressing critical safety concerns.^6,7^

SSEs are broadly categorized into four groups based on their distinct chemical composition: inorganic solid electrolytes (ISEs),^8^ polymer solid electrolytes (SPEs),^9–11^ polymerized ionic liquids (PILs),^12,13^ and composite solid-state electrolytes (CSEs). Regarding the ISEs, mainly oxide-type, sulfide-type, and sodium conductors have gained much attention. Although some of them deliver a high ionic conductivity at room temperature, ISEs still suffer from several challenging issues, including low Coulombic efficiency, low specific discharge capacity, poor rate performance, or poor cycling performance, resulting from their rigid nature.^14^ Furthermore, when sulfide ISEs are exposed to air, hazardous for humans, H_2_S can be released. In contrast to ISEs, polymer-based electrolytes have favorable properties, including low flammability, size flexibility, light weight, ease of processing, good interfacial contact, and electrode compatibility.^15^ Most SPEs are fabricated from lithium salts dissolved in ion-solvating polymers, such as poly­(ethylene oxide) (PEO), propylene oxide, poly­(ethylene imine), and polyalkene sulfides.^16–18^ Polymerized ionic liquids (PILs) attempt to bridge this gap by combining polymeric frameworks with IL moieties, but their single-ion conduction mechanism inherently limits charge transport.^19–21^ Since the conducting and mechanical properties are mutually exclusive, i.e., an enhancement in conductivity is achieved at the expense of reduced mechanical strength and vice versa, so far, very few PILs can satisfy all the industrial requirements. Multiphase composites, on the other hand, offer an opportunity to compromise on these often-conflicting requirements.^22,23^

Recent advances in electrolyte design have leveraged precisely engineered nanostructures to overcome traditional material limitations.^24,25^ Among these, polyhedral oligomeric silsesquioxanes (POSS) offer unprecedented molecular control; their cubic silica cores (1–3 nm) with eight strategically functionalized vertices enable programmable interactions with ionic species. The diversity of organic substituents situated at the corners of the POSS cage provides unique surface chemistry, leading to noncovalent interactions with the ions and, thus, the formation of three-dimensional networks, improving the mechanical properties and thermal stability of composites.^26^ Consequently, unlike conventional nanofillers, POSS nanoparticles create well-defined percolation pathways through their stoichiometric charge distribution, simultaneously enhancing mechanical robustness.^27^ As an example, it has also been shown that the addition of NH_2_-functionalized POSS particles to an [N_2288_]­[TFSI] ionic liquid leads to fast cation motions through the POSS-TFSI network, a charge transport mechanism similar to that observed for rigid PILs. As a consequence of the decoupling between the time scale of charge transport (determined by anion motions) and the structural relaxation (governed by the POSS-TFSI network), dc-conductivity (σ_dc_) at the liquid–glass transition temperature (*T*_g_) was three orders higher compared to pure aprotic ionic liquids. However, at the same time, the σ_dc_ at room temperature conditions was two decades below that of a pure ionic liquid.^28^ This result is simple to comprehend if we keep in mind that σ_dc_ is directly related to the number of ions, their charge, and mobility (σ_dc_ = *N*·*z*·μ). Consequently, when nonionized components are added to an ionic liquid, the quantity of ions per volume unit falls, lowering σ_dc_. In this regard, the incorporation of ionized POSS particles is expected to improve not only the mechanical properties but also the electric conductivity of ILs.

Herein, to verify this hypothesis, two types of POSS nanoparticles were selected: octaamonium POSS (C_24_H_72_Cl_8_N_8_O_12_Si_8_,), commercially known as AM0285, and PSS hydrate-octakis­(tetramethylammonium) substituted, abbreviated as TMA-POSS. The chemical structures of both nanofillers are depicted in Figure 1. As presented, AM0285 contains eight positively charged arms with NH_3_^+^ groups and the same number of small chloride counterions. In contrast, the core of TMA-POSS is negatively charged with ammonium counterions, [N_1111_]^+^. Consequently, the single silica nanoparticle carries a charge 8-fold larger compared to typical ions in ILs and potentially can bring an increase of σ_dc_. Since the final value of σ_dc_(RT) for the nanocomposite will be limited by interactions between the charged POSS core and ions of the matrix, it is crucial to select the proper ionic liquid enabling effective dissociation of counteranions. For this purpose, representatives of imidazolium-, pyrrolidinium-, and ammonium-based ILs were chosen. Specifically, among the pyrrolidinium ILs, [BMPyrr]­[TFSI], [BMPyrr]­[TFO], and [BMPyrr]­[TCM] were examined. Ammonium ILs are represented by materials containing a [TFSI]^−^ anion and [N_2228_]^+^, [N_1888_]^+^, and [N_122(2O1)_]^+^ cations. On the other hand, [BMIm]­[TCM] and [BMIm]­[BETI] fluids belong to the imidazolium class. The selected ILs feature butyl substituents (e.g., [BMPyrr]^+^, [BMIm]^+^) because, unlike ILs with longer or shorter alkyl chains, they exhibit a negligible crystallization tendency in the supercooled state. Only ILs with the [BMPyrr]^+^ cation crystallize when reheated from the glassy state. At the same time, anions like [TFSI]^−^ and [TCM]^−^ were chosen for their high electrochemical stability and ability to dissociate in the presence of charged POSS nanoparticles. The chemical structures of all of the examined ionic liquids and nanofillers are presented in Figure 1.

**Figure 1:**
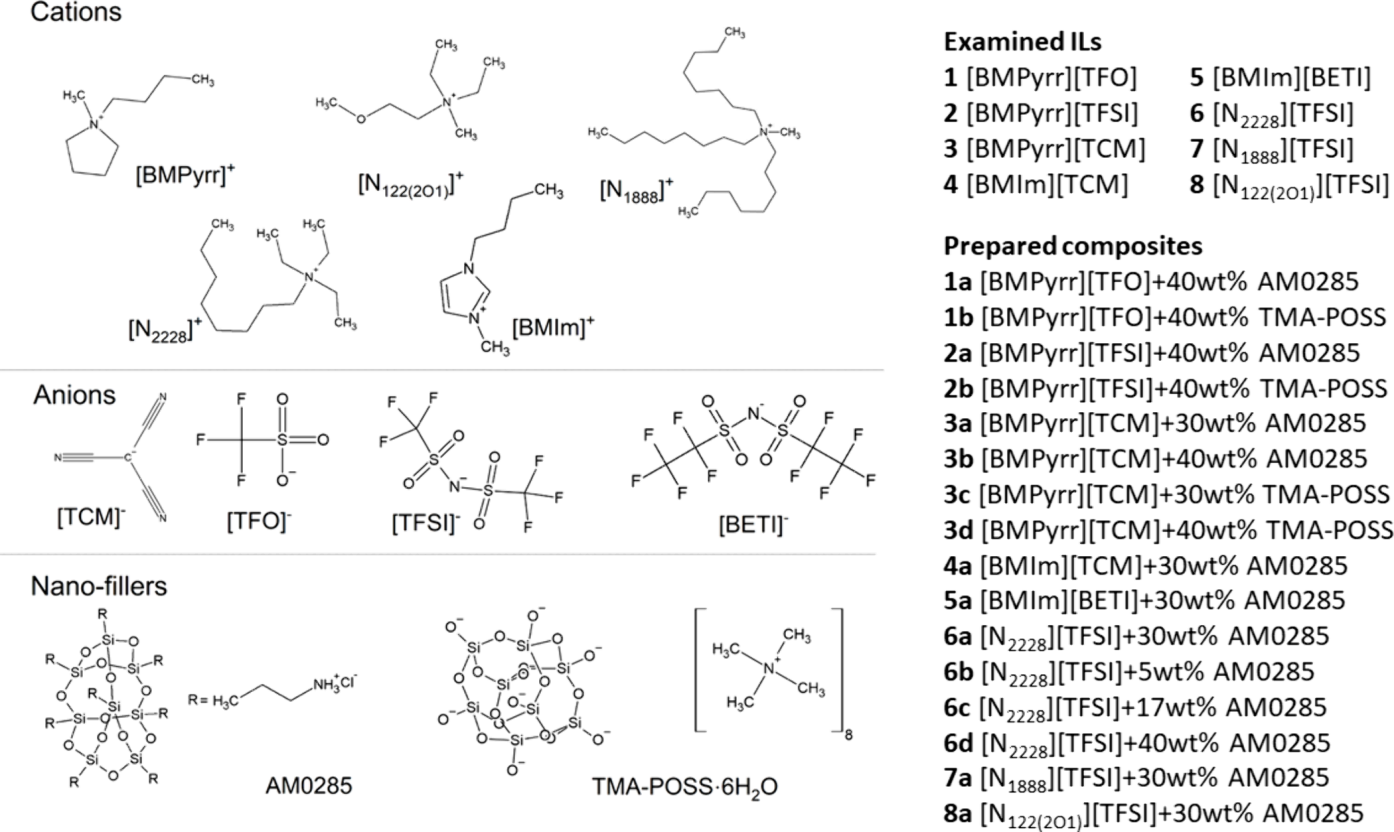
Chemical structures of the electrolyte components. The full names of cations and anions are given in the SI file.

The composites were obtained by conventional mixing of an ionic liquid and nanofiller in a ratio that provides a solid-state electrolyte, i.e., 30 and 40 wt % of POSS powder. All 13 samples obtained are viscous homogeneous mixtures (see Figure 2a). For the initial characterization of obtained composites, temperature-dependent thermogravimetric analysis (TGA) was performed (details in the SI). The decomposition temperature and amount of water adsorbed during the sample preparation are given in Table S1. Since all the examined materials contain around 2% water, time-dependent TGA scans were performed to determine the proper drying protocol. We found that 1 h at 373 K is enough to remove the moisture from AM0285-based composites (note that both IL and AM0285 particles were initially dried; see the SI for details). At the same time, 3 h at 353 K was required to dry TMA-POSS-based systems. The longer drying time was due to the fact that pure TMA-POSS powder contains 9% water, which corresponds to 6 water molecules per single POSS particle. Since the TMA-POSS substrate is highly hygroscopic and chemically unstable during drying at temperatures above 353 K, the number of composites containing this nanofiller was limited to [BMPyrr]-based samples.

**Figure 2:**
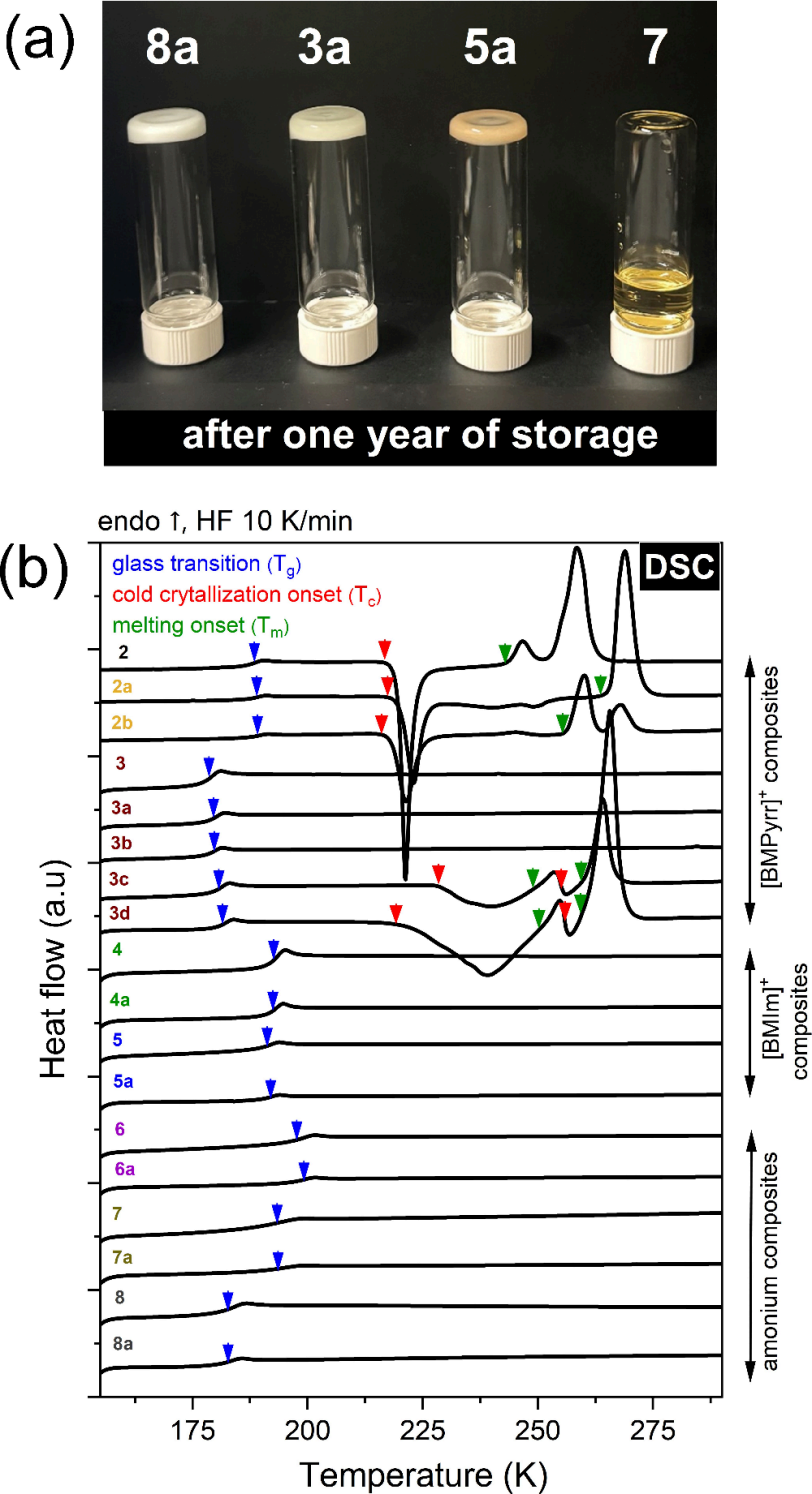
(a) Composites after one year of storage upside down. (b) DSC thermograms of ILs and their composites. Blue arrows indicate a liquid–glass transition; red arrows denote the onset of cold crystallization; and green arrows indicate the onset of melting. Numbers correspond to ionic liquids and composites listed in Figure 1.

The dried composites were next subjected to calorimetric measurements to determine their glass-forming ability and crystallization tendency. Almost all examined systems can be cooled to a glassy state without crystallization. The exceptions are composites containing [BMPyrr]­[TFO] (**1a**, **1b**); when they are anhydrous, they easily crystallize when the temperature decreases below 248 K (see Figure 2S). On the other hand, pyrrolidinium composites containing [TFSI]^−^ and [TCM]^−^ anions, specifically **2a**, **2b**, **3c**, and **3d**, crystallize on reheating from the glassy state (see Figure 2b). The presence of cold crystallization in composites based on [BMPyrr]­[TFSI] (no. 2 series) is rather natural due to the ordering tendency of a pure IL. However, depending on the type of NPs, different crystalline forms are obtained. On the other hand, for [BMPyrr]­[TCM]-based systems, POSS surface chemistry dictates whether IL ordering is partially preserved or fully suppressed. Specifically, in contrast to AM0285-based pyrrolidinium composites (**3a** and **3b**) that remain disordered over a broad temperature range, the addition of TMA-POSS NPs to [BMPyrr]­[TFSI] (**3c** and **3d**) evidently induces cold crystallization. This can be due to the rigid anionic core of TMA-POSS that interacts with pyrrolidinium cations.

From Figure 2b, it can also be noticed that the liquid–glass transition temperature of every examined composite is quite close to the *T*_g_ value of the corresponding ionic liquid (see Table S1). Since vitrification temperature is a key parameter controlling the conducting properties of electrolytes at room temperature conditions, higher *T*_g_, brings lower σ_dc_(RT), one can expect that prepared composites reveal relatively high σ_dc_(RT). To verify this statement, the dielectric data of pure IL and IL-NPs composites were gathered over a wide temperature range.

The representative dielectric results obtained for composite **4a** containing [BMIm]­[TCM] and 30 wt % of AM0285 nanoparticles are presented in a conductivity formalism in Figure 3a. The conductivity spectra σ′(*f*) measured over eight decades of frequency (10^–1^–10^7^ Hz) in a wide temperature range exhibit the behavior typical for ionic materials with an ac-conductivity at higher frequencies, followed by a dc-conductivity plateau σ_dc_ and a decrease of σ′ from σ_dc_ denoting the electrode polarization. From further inspection of temperature-dependent σ′(f) data, it becomes evident that isobaric cooling brings about a dramatic decrease in the dc-conductivity value, which is a quasi-universal behavior.^29,30^ Therefore, to describe the ion dynamics in IL-NPs composites more quantitatively, the temperature evolutions of σ_dc_ were determined. The log σ_dc_ vs inverse of temperature for representative pyrrolidinium, imidazolium, and ammonium composites are depicted in Figure 3b. As recognized here, log σ_dc_(1000*T* ^–1^) dependences reveal two characteristic regions separated by the liquid–glass transition (*T*_g_). Above *T*_g_, i.e., in the supercooled liquid state, the dc-conductivity follows the non-Arrhenius behavior, while below *T*_g_ (see the dashed line in Figure 3b), it can be described by the Arrhenius equation. For composite **4a** and the related [BMIm]­[TCM] IL, this effect is the most noticeable. However, an essential outcome coming from Figure 3b is that the dc-conductivity of examined IL-NPs mixtures determined at room temperature conditions, defined here as 298 K, is very close to that of pure IL. To visualize this effect, log σ_dc_ values of all examined ILs and their nanocomposites measured at 298 K were compiled in Figure 3c.

**Figure 3:**
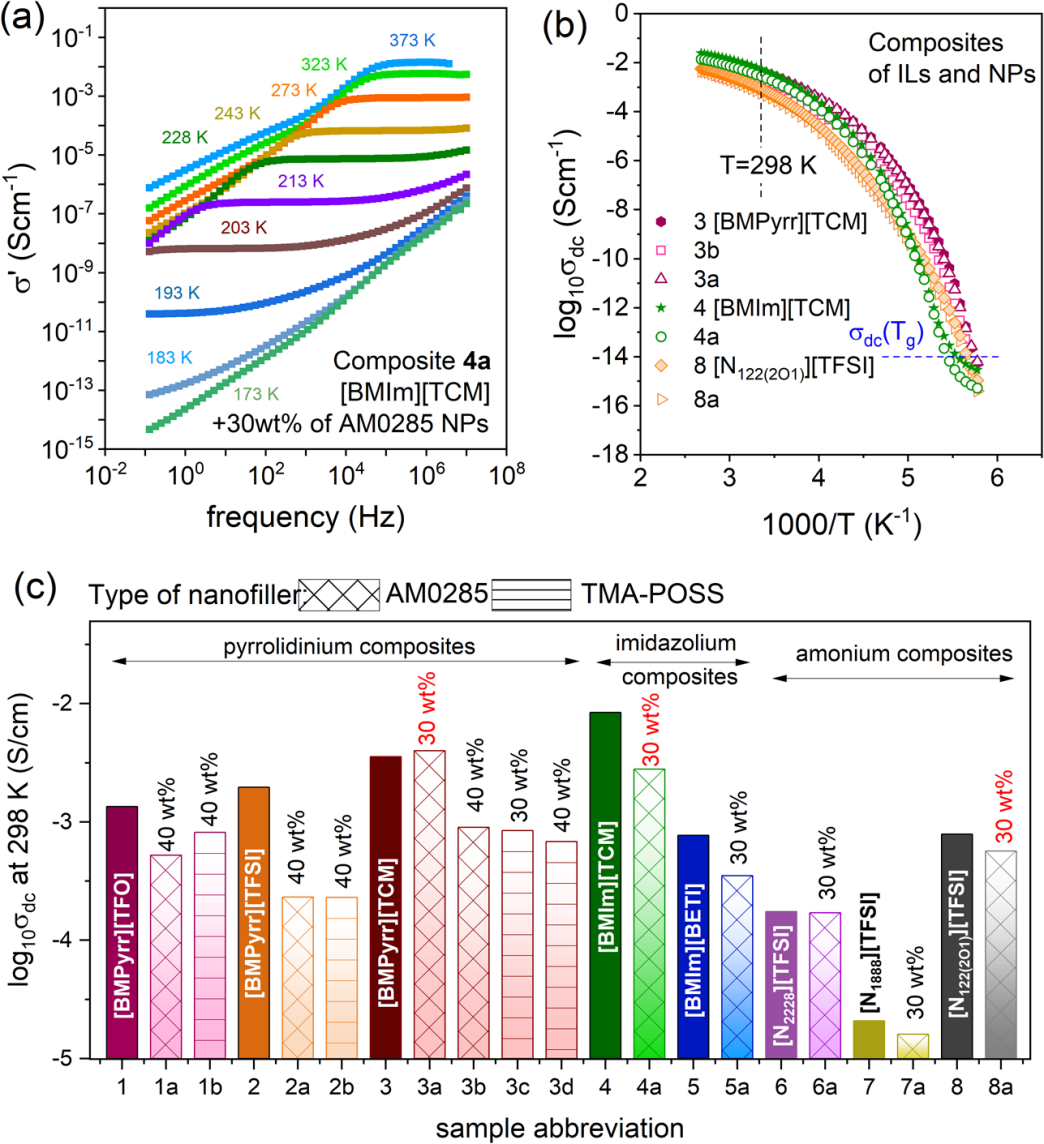
(a) The representative conductivity spectra of composite **4a** collected over a broad temperature range. The frequency-independent part denotes the dc-conductivity region. (b) Temperature dependence of log_10_ σ_dc_ of selected ILs and their composites. The dashed blue line indicates a dc-conductivity of 10^–14^ S/cm that usually corresponds with the conductivity value at *T*_g_. (c) log_10_ σ_dc_ determined at 298 K for all of the examined systems herein. Red numbers indicate composites revealing the highest conductivity within the pyrrolidinium, imidazolium, and ammonium ILs.

Overall, the σ_dc_(RT) of quasi-solid-state nanocomposites is lower than that of the corresponding IL determined under the same *T* conditions. An exception from this observation is composite **3a**, which shows a slight increase in dc-conductivity compared to the [BMPyrr]­[TCM] ionic liquid. The conducting properties of the **4a** composite, although worse than those of its parent IL, are similar to those of the [BMPyrr]­[TCM]-based compound (**3a**). At the same time, among the ammonium composites, the RT conductivity of **6a** shows quite similar conductivity to its parent IL. To recognize the molecular origin of this observation, we performed the Raman measurements of **3a**, **4a**, and **6a** composites (see the SI for details). The pure [BMPyrr]­[TCM], [BMIm]­[TCM], [N_2228_]­[TFSI], and AM0285 NPs were examined as a reference. The Raman data (Figures 3S–5S) reveal fundamental differences in how ionic liquid components interact with AM0285 nanoparticles depending on the cation structure. For [BMIm]­[TCM], the imidazolium cation shows indirect interactions with NPs primarily through weak hydrogen bonding between the terminal −CH_3_ groups of the flexible butyl chain (retaining G-conformers) and NP siloxane sides (Si–O···H–C). At the same time, [TCM]^−^ anions experience only minor perturbations (2 cm^–1^ C≡N upshift) and partial liberation (new 2220 cm^–1^ peak). This creates a fluid composite with mobile anions. In contrast, the [BMPyrr]­[TCM] system exhibits stronger interfacial organization: the butyl chain of the pyrrolidinium cation undergoes a drastic conformational shift, with G-type conformers (GG/GA) nearly disappearing and A-type conformers (AG/AA) dominating. This suggests rigid, extended chain packing near the NP surface, leading to a more structured interfacial layer around the NPs. This time, [TCM]^−^ anions show significant spectral changes: −C≡N band splitting with new band arround 2137 cm^–1^, and downshifting (from 2194 to 2159 and from 2218 to 2203 cm^–1^) indicating hydrogen bonding between [TCM]^−^ nitrile groups (−C≡N) and NP surface protons (−NH_3_^+^) and thus charge transfer with NP surfaces. These cation-dependent mechanisms create distinct nanocomposite morphologies: [BMIm]-based systems maintain ionic mobility through weak, alkyl-dominated interactions, whereas [BMPyrr] systems form structured interfacial layers through hydrogen-bonding networks, enhancing surface charge transfer. The findings demonstrate how cation selection (flexible imidazolium vs rigid pyrrolidinium) controls NP-IL interfacial design, enabling tunable properties for applications ranging from energy storage to functional nanocomposites.

In analogy to the [BMPyrr]­[TCM] composite, in **6a** the anion (this time [TFSI]^−^) interacts through hydrogen bonding with the ammonium groups on NPs (N–H^+^···O=S, the new 745 cm^–1^ band). The downshift and broadening of the 739 cm^–1^ “breathing mode” indicate in turn weakened [N_2228_]^+^···[TFSI]^−^ ion pairing due to conformational changes within the anion. . The [N_2228_]^+^ cation likely interacts indirectly through steric hindrance as its aliphatic chains disrupt anion–cation coordination without strong binding to NPs. Overall, NPs promote decoupled [TFSI]^−^ anions and locally ordered ionic domains, enhancing the interfacial charge heterogeneity.

While this study primarily examines 30–40 wt % POSS composites to demonstrate quasi-solid electrolyte functionality, we extended our investigation to lower loadings (5–20 wt %) to assess practical applicability. Systematic studies of [N_2888_]­[TFSI] with 5, 17, and 40 wt % AM0285 (presented in the SI file) reveal two key trends: (1) enhanced room-temperature conductivity (up to 17 wt %, Figure 6S) compared to pure IL, suggesting preserved ion mobility at reduced POSS content; and (2) loss of self-supporting mechanical integrity below 30 wt % (see mechanical data in the SI, Figure 8S), confirming the critical percolation threshold for quasi-solid behavior. This trade-off highlights the need for application-specific optimization between ionic transport and mechanical stability.

In the next step, mechanical measurements were performed to determine the extent to which the conducting and viscoelastic properties of the studied nanocomposites are coupled to each other. Since nanodispersions usually exhibit complex rheological properties, two types of experiments were performed: (i) dynamic (oscillatory) shear tests and (ii) steady shear experiments. The former can provide insight into structural relaxation and dynamic heterogeneity in ionic nanocomposites, while the latter is crucial to material verification before production and utilization on a larger scale.

The mechanical data of representative IL [BMIm]­[TCM] and its corresponding nanocomposite (**4a**) recorded at various temperature conditions in the vicinity of the liquid–glass transition are presented in the form of master plots in Figures 4a and 4b. Analogous data obtained for composite **3a** and the [BMPyrr]­[TCM] ionic liquid are included in Figure 7S. As depicted, the imaginary component of mechanical modulus *G*″(*f*) takes the form of the well-resolved peak with the *G*′–*G*″ crossover defining the structural relaxation time τ_α_. Note that *G*′(*f*) and *G*″(*f*) functions cross each other in *G*″ maximum only for pure IL. Furthermore, the loss modulus spectra *G*″(*f*) of the nanocomposite are much broader compared to IL, reflecting the heterogeneous nature of the structural dynamics. Another noticeable difference concerns the behavior of complex viscosity, which reveals the plateau range for IL and is frequency-dependent for the composite. Since η­(*T*) dependence of the nanocomposite cannot be determined directly from the viscosity plateau, as for IL, it was calculated by using the Maxwell relation η = τ_α_ *G*_∞_. From Figure 4c, it becomes evident that in the close vicinity of the liquid–glass transition, the η of the nanocomposite is slightly larger than that of pure IL. This result corresponds well with the higher *T*_g_ of nanofluid **4a** when compared to IL. Note that above 233 K, the η­(*T*) data are unavailable for composite since the time–temperature superposition rule is no longer obeyed. This suggests that the examined nanofluid starts to obey non-Newtonian behavior. Therefore, in the next step, we performed the shear rate experiments at 0.01 to 800 s^–1^ and at a few different temperatures.

**Figure 4:**
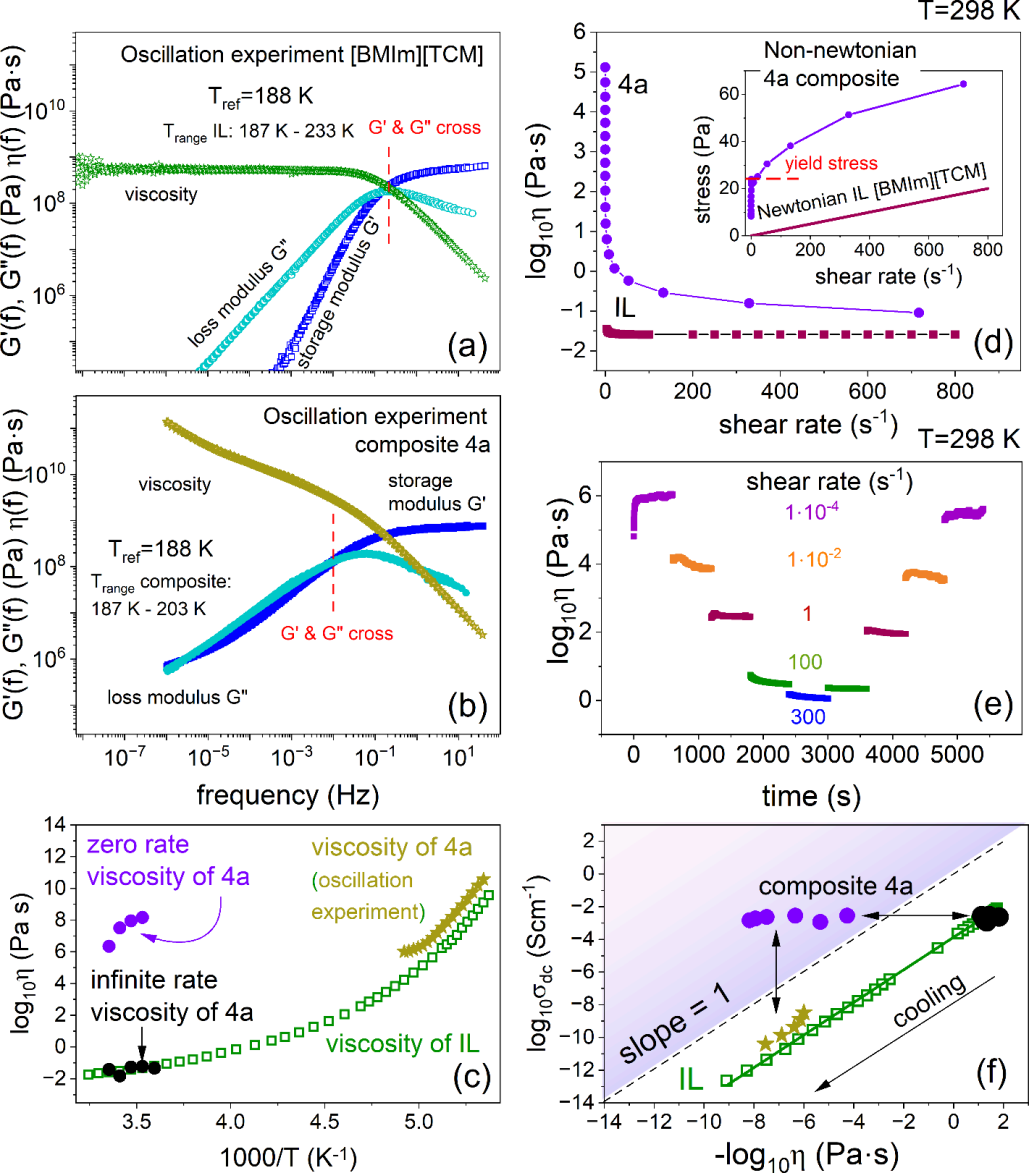
(a) Real *G*′(*f*) (blue symbols) and imaginary *G*″(*f*) (cyan symbols) parts of the complex shear modulus and complex viscosity (green symbols) of the pure [BMIm]­[TCM] IL collected over the temperature range 187–233 K and superimposed to the spectra measured at 188 K. (b) Real *G*′(*f*) and imaginary *G*″(*f*) part of the complex shear modulus and complex viscosity of composite **4a** collected over the temperature range 187–203 K and superimposed to the spectra measured at 188 K. (c) The comparison between complex viscosity determined for pure IL and nanofluid **4a**. Green squares indicate viscosity data obtained for IL [BMIm]­[TCM]. Violet circles denote zero rate viscosity of nanofluid **4a**, while black circles indicate infinite rate viscosity of composite **4a**. Stars denote the viscosity of composite **4a** determined from the oscillation experiment close to the liquid–glass transition. (d) Viscosity as a function of shear rate for composite **4a** and the corresponding ionic liquid measured at 298 K. The inset shows the stress vs shear rate graph. (e) Viscosity curves as a function of time and different shearing rates for composite **4a**, exhibiting its thixotropic behavior. (f) Walden plot constructed for the IL ([BMIm]­[TCM], green squares) and composite **4a**. The meaning of violet circles, black circles, and stars is the same as in (c). The reversible shear thinning is depicted.

Figure 4d shows the apparent viscosity and stress behavior as a function of the shear rate for the **4a** nanofluid and the corresponding IL as a reference. The obtained experimental data indicated that a pure IL is a simple Newtonian fluid with constant shear-independent viscosity. On the other hand, the addition of AM0285 nanoparticles results in a much more viscous system with significant shear-thinning properties. Namely, the apparent viscosity at zero rates, i.e., the viscosity of the material when it is effectively at rest, is almost seven decades larger than that of the base fluid. In contrast, the infinite rate viscosity equals η of the IL at the same *T* conditions (see Figure 4c). This behavior probably results from the disruption of the NP-IL dynamic network, described above, at higher shear rates.^31^ From the inset of Figure 4d, it can also be noticed that the stress experienced by the nanofluid is related to the shear rate in a nonlinear way, with the yield stress of 25 Pa quantifying the stress that the composite may experience before it begins to flow. Thus, the stress vs shear rate plot of nanofluid **4a** follows the Herschel–Bulkley behavior.

To recognize whether the shear thinning properties of the examined nanofluid are reversible, we monitored viscosity changes over time as a function of the shear rate. Figure 4e shows the experimental results obtained by varying the shear rate gradually, starting from a low value of 10^–4^ s^–1^, increasing to a shear rate of 300 s^–1^, and then decreasing to the initial value. As can be seen, the dynamic viscosity decreased with increasing shear rate and subsequently increased as the shear rate decreased. Furthermore, it takes a relatively short time to attain equilibrium viscosity when introduced to a steep change in shear rate. This indicates that the examined nanofluid reveals thixotropic behavior, i.e., the dynamic POSS-IL network providing efficient dc-conductivity rebuilds over time. Note that analogous results obtained for composite **3a** are shown in the SI file. Herein, note that phase separation was not observed for any of the examined nanocomposites after mechanical measurements.

Finally, to quantify to what extent the conducting and viscoelastic properties of the studied nanocomposite are coupled to each other, the Walden plot has been constructed. Figure 4f represents the Walden graph, with the “ideal” line (dashed line) corresponding to the case of complete coupling of ion conductivity to structural dynamics. Our data demonstrate that in the examined IL [BMIm]­[TCM], the conductivity entirely depends on viscosity, as the slope is the same as that of the ideal case. At the same time, the data below the ideal line indicate a poor dissociation of ion pairs. Such behavior is in line with most aprotic ILs, which show vehicle-type conduction. On the other hand, a high concentration of POSS nanoparticles substantially increases the viscosity while maintaining the conducting properties of the liquid state. Consequently, nanofluid **4a** is located above the ideal Walden line; that is, its viscosity is decoupled from dc-conductivity. This result could stem from modified ion dissociation or POSS-induced percolation pathways; however, future studies (e.g., NMR) are required to fully resolve the mechanism. From Figure 4f it can also be observed that application of the shearing force temporarily decreases the viscosity of the composite while keeping the conducting properties at the same level. This makes the obtained nanocomposites easily moldable.

In conclusion, we have demonstrated a novel class of quasi-solid electrolytes based on ionic liquids (ILs) and multiply charged polyhedral oligomeric silsesquioxane (POSS) nanoparticles that overcome the fundamental trade-off between ionic conductivity and mechanical stability in solid-state electrolytes. The NH_3_R^+^Cl^–^-functionalized POSS nanoparticles serve as both structural scaffolds and ionic conductors, enabling the formation of nanocomposites with exceptional properties. The optimal 30 wt % POSS composites exhibit ionic conductivities up to 4 mS/cm at room temperature, matching or exceeding their parent ILs, while maintaining quasi-solid-like mechanical integrity and thixotropic processability. This achievement addresses a long-standing challenge in electrolyte design, where the conductivity typically plummets upon solidification. Unlike conventional filters that trap ions, our charged POSS nanoparticles create percolating ion channels that depend on cation selection, while simultaneously providing structural reinforcement. The decoupled ion transport and reversible shear-thinning behavior represent a significant advance over prior POSS-IL composites, which showed either conductivity losses or irreversible aging. The obtained nanofluids are thermally and physically stable without any crystallization or phase separation even one year after preparation, which makes them promising for practical application.

## Supplementary Material



## Notes

All experimental details and supporting results of DSC, TGA, Raman and mechanical (viscosity) experiments (PDF)
