# Supplementary material for: Tuning Ionic Liquids with Charged Polyhedral Oligomeric Silsesquioxane Nanoparticles for Highly Conductive Quasi-Solid Electrolytes
Source: Nano Lett. 2025 Jun 6;25(24):9779–86. doi: 10.1021/acs.nanolett.5c02053 (PMC12186608; doi:10.1021/acs.nanolett.5c02053)
Supplement: Supplementary file 1 [file nl5c02053_si_001.pdf]

Supporting Information

Tuning Ionic Liquids with Charged Polyhedral Oligomeric Silsesquioxane Nanoparticles for Highly Conductive Quasi-Solid Electrolytes

Soorya Koymeth[1], Marian Paluch[1], Mateusz Dulski[2], Zaneta Wojnarowska[1]

[1]*Institute of Physics, the University of Silesia in Katowice, 75 Pułku Piechoty 1A, 41–500 Chorzów, Poland*

[2]*Institute of Material Sciences, the University of Silesia in Katowice, 75 Pułku Piechoty 1A, 41−500 Chorzów, Poland*

Experimental

Examined samples. The examined ionic liquids were purchased from IoLiTec (Ionic Liquids Technologies GmbH). 1-Butyl-1-methylpyrrolidinium triflate (BMPyrr][TFO] 99% (Molecular weight: 291.33 g/mol); 1-Butyl-1-methylpyrrolidinium bis(trifluoromethylsulfonyl)imide, 99.5% [BMPyrr][TFSI] (Molecular weight: 422.41), 1-Butyl-1-methylpyrrolidinium tricyanomethanide, 98% ([BMPyrr][TCM] (Molecular weight: 232.32 g/mol), 1-Butyl-3-methylimidazolium tricyanomethanide, 98% ([BMIm][TCM]) (Molecular weight: 229.28 g/mol), 1-Butyl-3-methylimidazolium bis(pentafluoroethylsulfonyl)imide, 98% ([BMIm][BETI]) (Molecular weight: 519.37 g/mol), Octyltriethylammonium bis(trifluoromethylsulfonyl)imide, >98% ([N_{2228}][[TFSI]) (Molecular weight: 494.56 g/mol), Methyltrioctylammonium bis(trifluoromethylsulfonyl)imide, 99% ([N_{1888}][[TFSI]) (Molecular weight: 648.85 g/mol), N,N-Diethyl-N-methyl-N-(2-methoxyethyl) ammonium bis(trifluoromethylsulfonyl)-imide, 99% ([$N_{122(2O1)}$][TFSI]) (molecular weight 426.40 g/mol). The ILs were dried at 353 K over 12 hours before preparation of composites. The water content determined by Karl-Fisher titration is provided in Table S1.

Octaamonium POSS with the molecular formula $C_{24}H_{72}Cl_8N_8O_{12}Si_8$ and molecular mass of 1173.18 g/mol, commercially known as AM0285®, was purchased from Hybrid Plastics.

PSS hydrate-Octakis(tetramethylammonium) substituted ($C_{32}H_{96}N_8O_{20}Si_8 \cdot x\ H_2O$; molecular weight 1137.83 g/mol (anhydrous basis)), abbreviated as TMA-POSS, was purchased from Sigma Aldrich. Drying of TMA-POSS powder at 353 K revealed a mass loss of 9%, which corresponds to 6 water molecules per single POSS particle.

Methods

Differential Scanning Calorimetry (DSC)

Thermal characterization of the functionalized POSS systems was conducted using a high-sensitivity differential scanning calorimeter Mettler Toledo DSC1STAR featuring a 120-thermocouple ceramic detection array. The instrument's cryogenic cooling system maintained precise temperature control throughout measurements, with a constant 60 mL/min inert gas purge ensuring oxidative stability. Calibration protocols employed high-purity metal references to verify both thermal response and energy quantification accuracy. For data analysis, linear baselines were algorithmically generated between transition boundaries, while integrated

software tools enabled comprehensive thermal event characterization, including phase transition energetics and temperature parameters.

Thermogravimetric Analysis (TGA)

Thermogravimetric analysis of the hybrid materials was conducted using a high-precision Mettler-Toledo, Switzerland microbalance system coupled with a programmable heating furnace. Samples (2-5 mg) were loaded into vented aluminum crucibles and subjected to controlled thermal ramping (10°C/min) under continuous nitrogen atmosphere (30 cm^3/min flow rate). Mass variations were monitored with ±0.1 μg resolution, with decomposition profiles quantified through differential mass loss calculations relative to initial sample weights.

Dielectric Measurements

The dielectric measurements at ambient pressure for studied FOS were carried out over a frequency range from 10^{-1} Hz to 10^{7} Hz by means of a Novo-Control GMBH Alpha dielectric spectrometer. The Novocool system controlled the temperature with an accuracy of 0.1 K. During this measurement, the sample was placed between two stainless steel electrodes (diameter = 15 mm). The quartz ring provided the distance between plates.

Viscosity measurements

The viscosity was measured employing an ARES G2 Rheometer. In the supercooled liquid region, aluminum parallel plates of diameter 4 mm were used, while the viscosity of normal liquid state was measured using 25 and 50 mm geometry. The rheological experiments were performed in the frequency range from 0.1 to 100 rad·s^{-1} (10 points per decade) with strain equal to 0.01% in the vicinity of the liquid glass transition. The strain was increased by one order of magnitude with every 10 K. The relative uncertainty of the reported viscosity measurements $u_r(\eta)$ from calibration, temperature and pressure control, as well as sample purities, did not exceed 7%.

Raman spectroscopy

Raman spectroscopy measurements were conducted on pure ionic liquids ([BMPyrr][TCM], [BMIm][TCM], and [N_{2228}][TFSI]), pure AM0285, and a series of IL–AM0285 composites. For $[N_{2228}]^+$-based composites various concentration of AM0285 NPs was used: 5, 17, 30, and 40 wt%. The measurements were performed using a WITec confocal alpha300 R Raman microscope equipped with an air-cooled solid-state laser (λ = 532 nm, power = 20 mW) and a CCD detector. The laser was coupled to the microscope via a polarization-maintaining single-mode optical fiber (50 μm core diameter) and focused onto the sample using an Olympus MPLAN objective lens (50×, NA = 0.7). Scattered Raman signals were collected through a multi-mode fiber of the same diameter. The spectrometer's monochromator (600 lines/mm grating) was checked using a silicon reference (520.7 cm^{-1}). To minimize statistical error arising from the nanoscale dimensions of the AM0285 filler and to accurately capture structural changes due to interactions between the ionic liquid and AM0285 functional groups, surface Raman imaging was performed in the X–Y plane over a 30 μm × 30 μm area. This high-resolution Raman map consisted of 90 × 90 pixels, generating a total of 8,100 individual spectra. Each spectrum was acquired with a 500 ms integration time, and sample positioning was

maintained with a precision of ±0.5 μm. Spectra were recorded over the 100–4000 cm^{-1} range at a spectral resolution of 3 cm^{-1}. Post-acquisition processing involved baseline correction using a fifth-degree automatic polynomial function and removal of cosmic ray artifacts, both carried out using WITec ProjectFive Plus software. The final dataset of surface imaging, comprising 8,100 spectra, was averaged to assess the structural modifications within the studied systems.

Supporting data

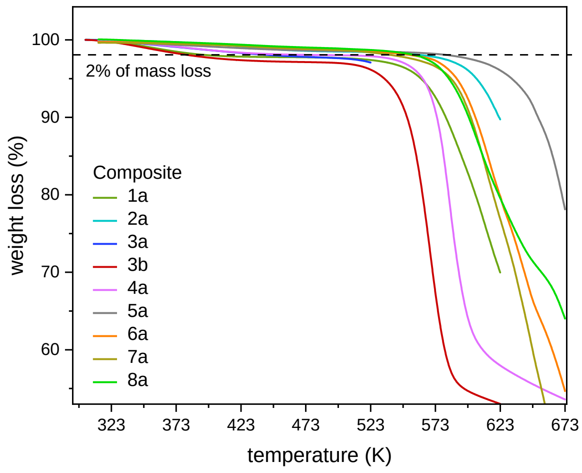

Figure 1S TGA curves of examined nanocomposites.

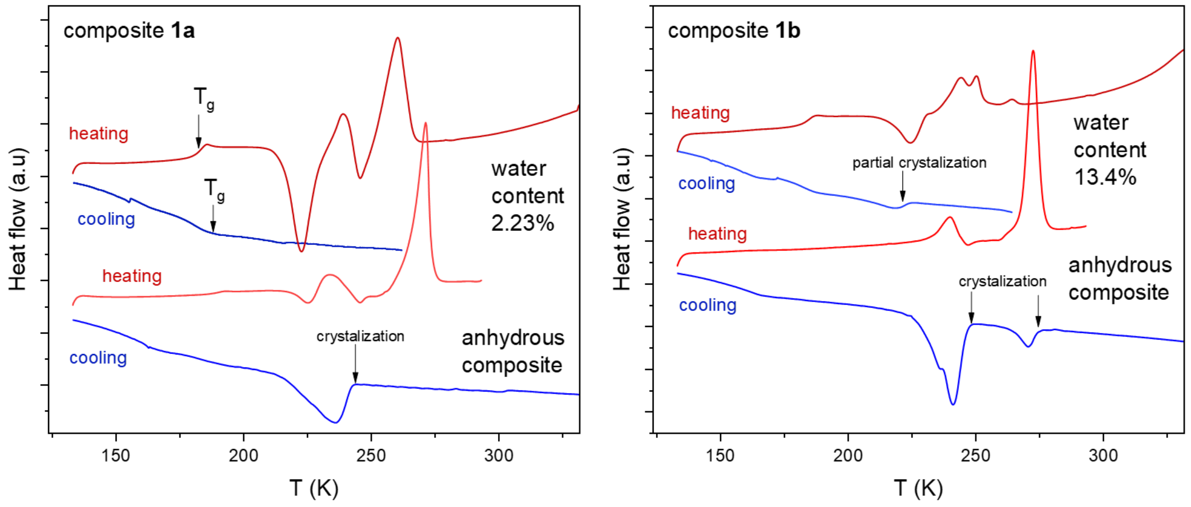

Figure 2S DSC scans of hydrated and anhydrous composites **1a** and **1b**.

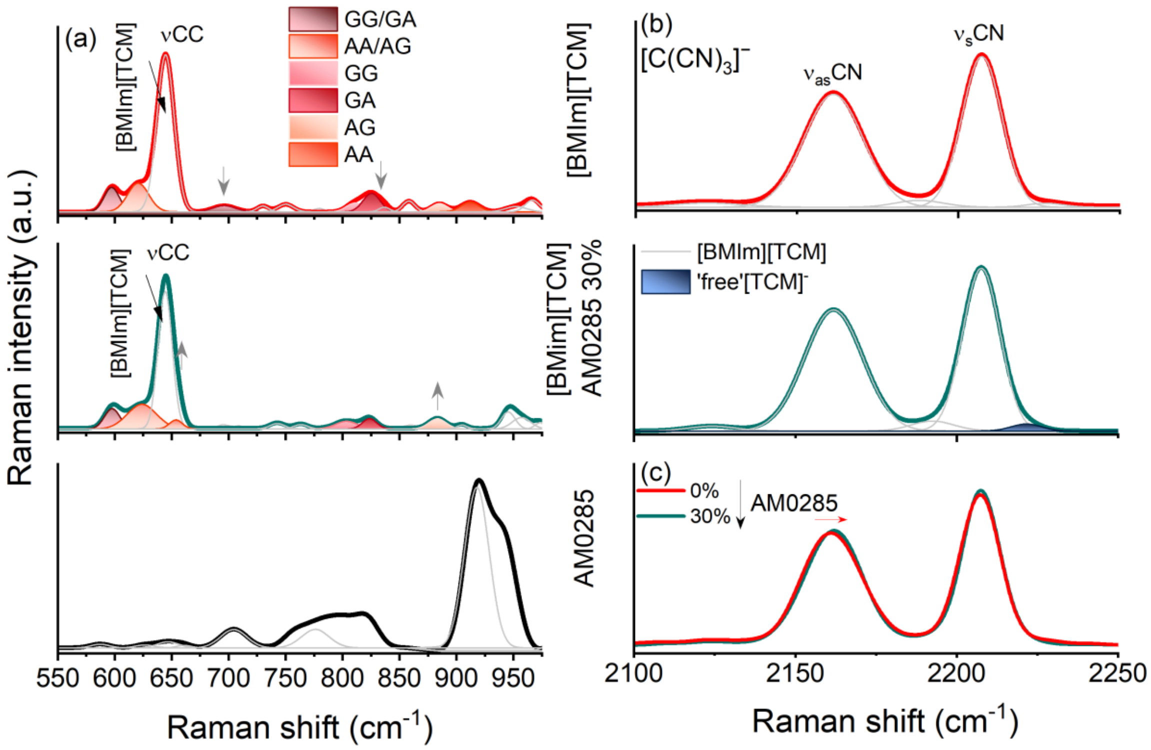

Figure 3S. Raman spectra of neat AM0285 filler, [BMIm][TCM] and composite containing 30% AM0285 filler are presented for the spectral ranges of 550–970 cm^{-1} (a) and 2100–3350 cm^{-1} (b). Additionally, the nitrile stretching region was summarized to compare the neat ionic liquid and the composite (c). Key spectral features corresponding to different conformational states of the butyl side chain (gauche–gauche, GG; gauche–anti, GA; anti–anti, AA; anti–gauche, AG) as well as the coordination environment of the $[TCM]^{-}$ counterion have been color-coded.

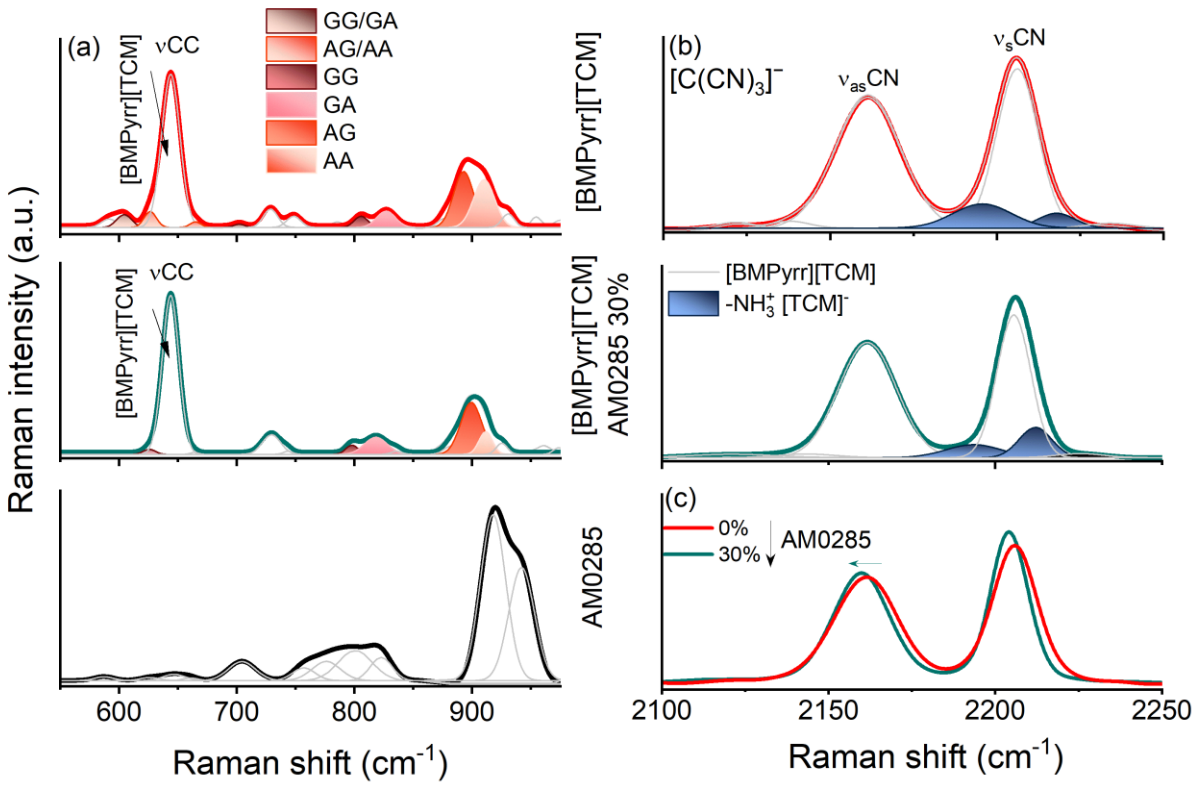

Figure 4S. Raman spectra of neat AM0285 filler, [BMPyrr][TCM] and composite containing 30% AM0285 filler are presented for the spectral ranges of 550–970 cm^{-1} (a) and 2100–3350 cm^{-1} (b). Additionally, the nitrile stretching region was summarized to compare the neat ionic liquid and the composite (c). Key spectral features corresponding to different conformational states of the butyl side chain (gauche–gauche, GG; gauche–anti, GA; anti–anti, AA; anti–gauche, AG) as well as the coordination environment of the [TCM]$^-$ counterion have been color-coded.

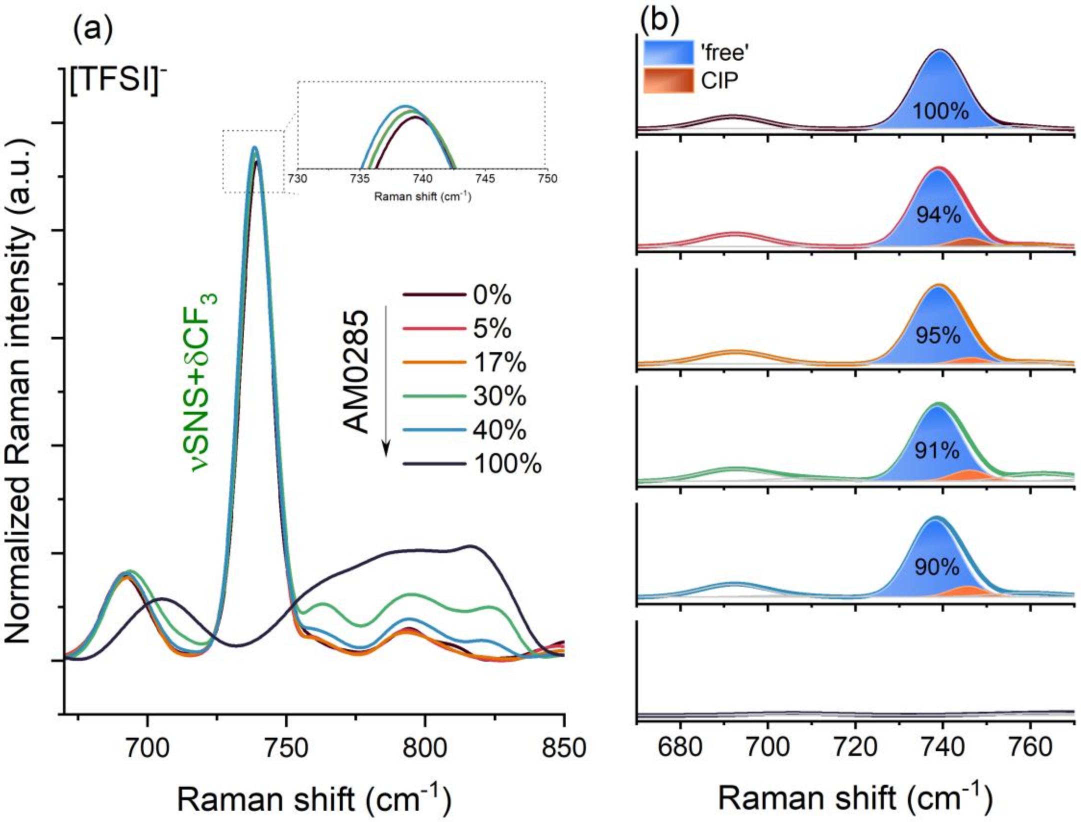

Figure 5S. Normalized Raman spectra of neat AM0285 filler, $[N_{2228}]$[TFSI] and composite containing 5%,17%,30%,40% AM0285 filler are presented for the spectral ranges of 670–850 cm^{-1} (a) and 670–770 cm^{-1} (b). Key spectral features corresponding to the coordination environment of the $[TFSI]^-$ counterion have been color-coded. CIP refers to contact ion pair.

Raman analysis was performed to evaluate the influence of the AM0285 filler on structural changes and molecular reorganization within ionic liquid (IL) composite systems (Figures S1–S3).

Composites based on [BMIm][TCM] and [BMPyrr][TCM]. Notably, the characteristic Raman band pattern of the tricyanomethanide anion remained largely unaffected, showing only minimal shifts, typically within 1 cm^{-1}, when comparing imidazolium- and pyrrolidinium-based systems (Figures 3S and 4S). In both cases, five prominent bands were consistently observed at approximately 152, 481, 644, 2161, and 2207 cm^{-1}, corresponding respectively to in-plane CCN bending, out-of-plane bending, symmetric CC stretching, and the antisymmetric and symmetric C≡N stretching vibrations[1,2,3].

After the incorporation of 30% AM0285 NPs to ILs with TCM anion, the CCN bending and CC stretching modes exhibited minimal sensitivity to structural variations. In contrast, the nitrile (C≡N) stretching vibrations were found to be more responsive to changes in the local chemical environment[4]. In composites containing $[BMIm]^+$ cation, a subtle yet consistent upshift of approximately 2 cm^{-1} of the nitryl-related bands suggests a structural reorganization of the ionic matrix upon interaction with the filler (Figure 3S a-c). This phenomenon can result from the presence of structural defects associated with the NPs, such as non-bridging oxygen

atoms, hydroxyl groups, or vacant coordination sites, which also may facilitate the partial decoupling of ion pairs, implying the formation of free or weakly associated $[TCM]^-$ anions. Some evidence of such an effect can be the appearance of an additional Raman component at around 2220 cm^{-1}, detected through spectral deconvolution (Figure 3S b-c). Additionally, an observed slight band upshift may result from conformational changes within the butyl side chain of $[BMIm]^+$, which promotes local rearrangement of the ionic network and enables new intermolecular interactions between the filler surface and the IL components[3].

In contrast to [BMIm]-composite, the Raman band profile of the antisymmetric and symmetric C≡N stretching modes in the $[BMPyrr]^+$-based ionic liquid reveals a more asymmetric shape, with additional bands observed at approximately 2137, 2194, and 2218 cm^{-1} (Figure 4S b). This complex band structure suggests a more intricate interaction network within the system. The weak band at 2137 cm^{-1}, as reported in the literature, is often associated with slight deviations from the ideal planar symmetry of the $[TCM]^-$ anion. These deviations may result from bending or tilting of the anion at the pyrrolidinium cation ring, which activates a second CN stretching mode. The other two additional bands (~2194 and ~2218 cm^{-1}) are attributed to the presence of free or weakly coordinated $[TCM]^-$ anions, which may interact with the cation through non-conventional bonding, possibly involving different conformations of the butyl side chain. However, upon the incorporation of the AM0285, the antisymmetric C≡N stretching mode becomes more symmetric, similar to the behavior observed in [BMIm][TCM] systems.

Additionally, the components initially associated with weakly bound or free $[TCM]^-$ anions were found to shift toward lower frequencies in composite. This downshift indicates enhanced intermolecular interactions, possibly driven by conformational rearrangements in the butyl chain that allow for closer packing or new interaction sites. An explanation for the observed downshift in the primary nitryl-related Raman bands from 2161 and 2207 cm^{-1} to 2159 and 2203 cm^{-1} (Figure 4S a–c) may involve direct interaction of the $[TCM]^-$ anion with ammonium protons originated from the NPs. This interaction could lead to partial charge delocalization within the anion structure. Compared to imidazolium or pyrrolidinium cations, the presence of positively charged ammonium groups has a more buried and less accessible positive charge center, which could stimulate the formation of more intermolecularly interacting systems. It leads to the degree of structural heterogeneity within the IL matrix, and it is often characterized by the coexistence of polar and nonpolar domains, as postulated earlier.[5,6] As with imidazolium-based systems, hydrogen bonding in the $[BMPyrr]^+$ systems may involve interactions between the nitrogen atoms of the nitrile groups or the central carbon of the $[TCM]^-$ anion with protons from ammonium groups, further contributing to the observed spectral modifications and structural reorganization.

The coexistence of the $[BMIm]^+$ cation with the $[TCM]^-$ anion in the ionic liquid state, as confirmed by both theoretical and experimental approaches, reveals that $[BMIm]^+$ adopts four distinct conformational states. These states are evidenced by characteristic Raman bands located at approximately 599 and 696 cm^{-1} (corresponding to GG and GA conformers) and 624 cm^{-1} (AG and AA conformers), as well as a second set of bands around 806 (GG), 827 (GA), 883 (AG), and 907 cm^{-1} (AA). These vibrations arise from combinations of ring-based HCCH and NC(H)N bending modes and stretching of the CC alkyl chain.[7,8] A similar vibrational pattern was observed for the $[BMPyrr]^+$ cation. However, upon cationic

substitution, the corresponding conformational bands are slightly shifted to higher frequencies, specifically to 606 and 700 cm^{-1} (GG and GA), 626 cm^{-1} (AG and AA), and to 805 (GG), 828 (GA), 890 (AG), and 912 cm^{-1} (AA).[9] Interestingly, the type of cationic ring plays a critical role in stabilizing specific conformational states and influences the overall organization of the ionic pairs. It was clearly marked by a notably stronger presence of bands corresponding to the AG and AA conformations of the butyl chain in the case of $[BMPyrr]^+$ compared to $[BMIm]^+$.

Upon incorporation of the AM0285 filler, the positions of the conformation-sensitive Raman bands remain essentially unchanged, indicating that the filler does not significantly alter the local vibrational environment. However, notable changes in band intensity reveal a structural reorganization of the alkyl chain. In the [BMIm][TCM] composite, a reduction in the intensity of bands associated with G-type conformers is observed (Figure 3S a), accompanied by a modest increase in bands related to A-type conformers. By contrast, the [BMPyrr][TCM] system exhibits a much more pronounced response. Here, the intensity of GG conformer bands significantly decreases or disappears entirely, while the AG and AA bands become dominant. It suggests that the AM0285 filler promotes selective stabilization of certain conformers within the butyl chain, depending on the cationic structure. A key factor influencing this behavior appears to be correlated with the hydrophilic nature of the $[TCM]^-$ anion. As a result, the ionic liquid network becomes more loosely packed, enabling enhanced conformational flexibility and favoring the formation of specific cationic conformers.

As a result, in the [BMPyrr][TCM] system there is a near-complete suppression of G conformers, while A-type conformers (AG and AA) dominate. This conformational shift likely promotes stronger interactions between the cation and the AM0285 surface. In contrast, in [BMIm][TCM] the presence of G-type conformers suggests possible hydrogen bonding between the terminal $–CH_3$ group of the butyl chain and the siloxane groups on the AM0285 surface. This interaction is consistent with a Si_2 –O···H–C hydrogen bond mechanism, as proposed in earlier studies.[10,11] Altogether, these findings emphasize that the structural reorganization of the alkyl chain in IL composites is highly dependent on the nature of both the cation and the anion. Similar effects have been observed in other ionic liquids where variation in anion size leads to changes in local order, confirming that structural heterogeneity and domain organization in IL systems are governed by subtle ion-ion and ion–surface interactions.[12]

Composites based on $[N_{2228}]$[TFSI]

The characteristic Raman spectrum of the bis(trifluoromethanesulfonyl)imide ($[TFSI]^-$) anion in the $[N_{2228}]$[TFSI] ionic liquid reveals the coexistence of cisoid (C1) and the transoid (C2) conformers. In the conformationally sensitive low-frequency region, vibrational bands attributed to the C1 conformer are observed at approximately 309, 324, and 408 cm^{-1}, while those associated with the C2 conformer appear at 293, 338, and 395 cm^{-1}. These bands arise from in-plane and out-of-plane vibrational modes of the SO_2 groups and reflect the distinct geometries of the anion.[13] Similar spectral patterns have been reported for $[TFSI]^-$ in the presence of different cations, confirming the conformer assignments.[14,15] In the mid-frequency region, weaker bands between 1350–1330 cm^{-1} and 1240–1190 cm^{-1} correspond to the antisymmetric and symmetric stretching vibrations of the SO_2 and CF_3 groups, respectively.

However, interpretation of this region is challenging due to overlapping signals from the cationic aliphatic chains, including CH_2 and CH_3 deformation modes, as well as CC and CN stretching vibrations of the cation. Hence, the most prominent and diagnostically useful feature in the Raman spectrum is a strong, well-resolved band centered around 739 cm^{-1}. This band results from a coupled vibrational mode involving CF_3 deformation and SNS stretching, commonly referred to as the "breathing mode" of the $[TFSI]^-$ anion (Figure 5S). Its position and intensity are reliable markers for identifying "free" $[TFSI]^-$ anions within the neat ionic liquid structure.

The "breathing mode" induces substantial changes in the polarizability of the anion, making them highly sensitive to variations in the local coordination environment.[16] As a result, detailed spectral fitting of these vibrational features provides critical insights into ion-ion and ion–polymer interactions. The position, shape, and intensity of the key bands are exceptionally responsive to changes in the anion's conformation[17] and coordination state[18], capturing subtle shifts in geometry, solvation, and local electrostatic effects. Hence, upon the addition of the AM0285 filler, the main Raman band of $[TFSI]^-$ located around 739 cm^{-1} undergoes a slight broadening and shifts to lower frequencies with increasing filler concentration (Figure 5S a). Simultaneously, a new band appears at approximately 745 cm^{-1}, whose intensity progressively increases as the filler content rises (Figure 5S b). These spectral changes can be interpreted through several overlapping effects.

One possible explanation for the observed spectral changes is a weakening of the coordination between the oxygen atoms of the $[TFSI]^-$ anion and the $[N_{2228}]^+$ cation. It may result from steric hindrance or thermal activation induced by the presence of the filler, which facilitates partial decoordination of the anion. In addition, the emergence of a new Raman band may indicate enhanced ion-ion and ion–polymer interactions, potentially resulting in the formation of dynamic ion–polymer networks or cross-linked structures.[19] As the concentration of AM0285 increases, the conformational equilibrium of the $[TFSI]^-$ anion appears to shift in favor of the trans conformer. This conformer is likely more stable in confined environments such as the pores of the silica filler. These effects are particularly relevant in regions of the silica surface containing structural defects such as non-bridging oxygen atoms or free silanol functionalities.[20,21] Finally, the observed conformational and spectral modifications suggest the formation of multidentate contact ion pair complexes involving hydrogen bonding between protonated ammonium groups and $[TFSI]^-$ anions. These interactions likely enhance intermolecular forces and support the development of locally organized ionic domains. The appearance of a Raman band at a similar frequency has previously been linked to a decrease in the population of "free" anions in systems containing chloride, reflecting a greater contribution from mixed anion contact ion pairs.[22]

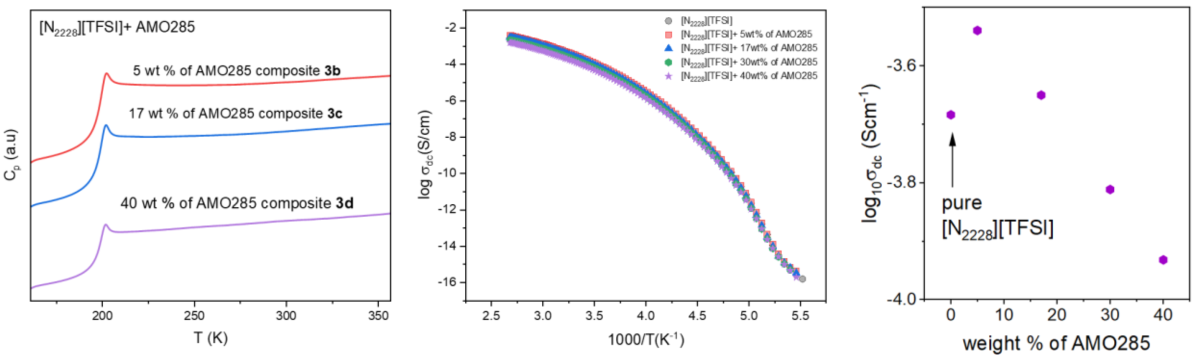

Figure 6S Left panel: DSC data of composites containing [N2228][TFSI] and various contents of AM0285 NPs. Middle panel: DC-conductivity data of [N2228][TFSI] and its composites measured over a wide temperature range. Right panel: dc-conductivity data determined at 298 K for ammonium composites.

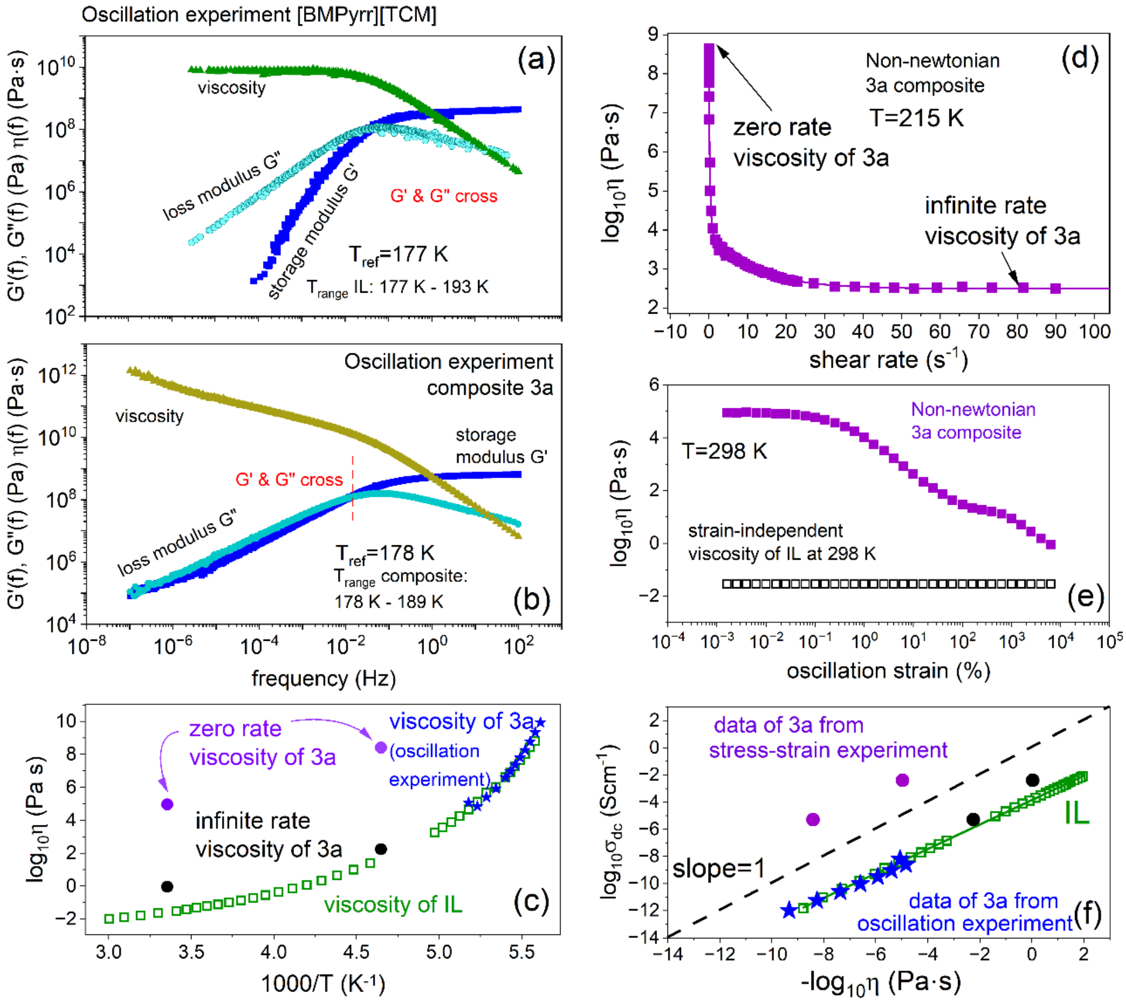

Figure 7S (a) Real G'(f) (blue symbols) and imaginary G"(f) (cyan symbols) part of the complex shear modulus and complex viscosity (green symbols) of pure [BMPyrr][TCM] IL collected over the temperature range 177-193 K and superimposed to the spectra measured at 177 K. (b) Real G'(f) and imaginary G"(f) part of the complex shear modulus and complex

viscosity of composite 3a collected over the temperature range 178-189 K and superimposed to the spectra measured at 178 K. (c) The comparison between complex viscosity determined for pure IL and nanofluid 3a. Green squares indicate viscosity data obtained for IL [BMPyrr][TCM]. Violet circles denote zero rate viscosity of nanofluid 3a, while black circles indicate infinite rate viscosity of composite 3a, determined at two different temperatures 215 K and 298 K. Stars denote the viscosity of composite 3a determined from the oscillation experiment close to the liquid-glass transition. (d) Viscosity as a function of shear rate for composite 3a measured at 215 K. Clear shear thinning behavior is visible. (e) Viscosity curves as a function of oscillation strain for composite 3a, determined at 298 K. Ionic liquid [BMPyrr][TCM], revealing Newtonian behavior, was examined as a reference. (f) Walden plot constructed for IL ([BMPyrr][TCM]-green squares) and composite 3a. The meaning of violet circles, black circles and stars is the same as in panel (c).

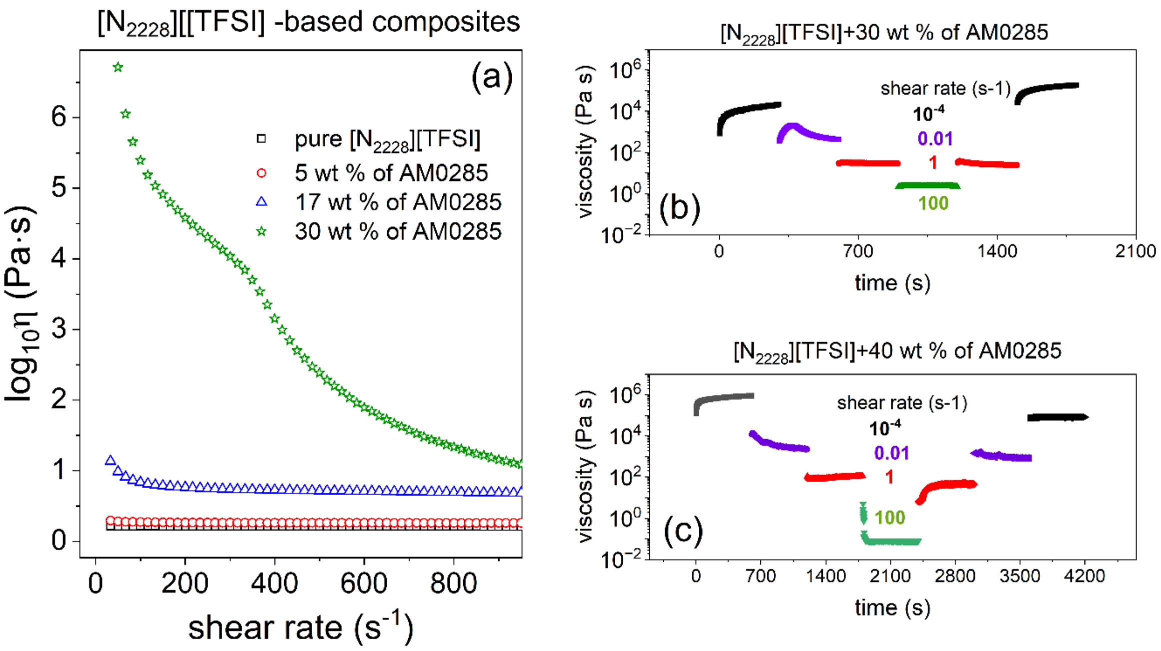

Figure 8S. (a) Viscosity as a function of shear rate for pure [N2228][TFSI] and composite containing different weight fraction of AM0285 NPs measured at 298 K. Clear shear thinning behavior is visible for composite containing 30 wt % of NPs. At the same time, composties with 5 and 17 wt % of NPs reveal Newtonian behavior. Panels (b) and (c) shows viscosity curves as a function of time and different shearing rates for composite containing 30 and 40 wt % of AM0285 NPs, exhibiting its thixotropic behavior.

Table S1 Physico-chemical characterization of examined mixtures. T_g- glass transition temperature, T_c-onset of crystallization, T_m-onset of melting point.

	IL type	Nanofiller	Weight % of NPs	Mole fraction x_{POSS}	N_{IL} per POSS molecule	T_g [K]	T_c [K]	T_m [K]	T decomposition [K]	Water content [%]	σ_{dc} at 298 K
**1**	[BMPyrr][TFO]					180.5[d]	210.9[d]	232.9[d]		0.27[b]	$8.61\cdot10^{-4}$
**1a**		AM0285	40	0.14	6	182.5[d]	208.9[d]<br>243.5[d]<br>243.5[e]	231.2[d]<br>249.5[d]	553	2.23[a]	$5.22\cdot10^{-4}$
**1b**		TMA-POSS	40	0.13	6.5	183.7[d]	210.5[d]<br>274.8[e]	230.4[d]<br>263.8[e]	373	13.4[c]	$8.17\cdot10^{-4}$
**2**	[BMPyrr][TFSI]					187.6[e]	216.6[e]	243.4[e]		0.11[b]	$8.10\cdot10^{-4}$
**2a**		AM0285	40	0.19	4	188.9[e]	217.0[e]	263.6[e]	591	1.45[a]	$2.31\cdot10^{-4}$
**2b**		TMA-POSS	40	0.18	4.4	189.2[e]	215.4[e]	255.7[e]	376	13.8[c]	$2.31\cdot10^{-4}$
**3**	[BMPyrr][TCM]					178.4[e]	-	-		0.1[b]	$3.5\cdot10^{-3}$
**3a**		AM0285	30	0.08	12	179.8[e]	-	-	524	2.21[a]	$4\cdot10^{-3}$
**3b**		AM0285	40	0.12	7.6	179.3[e]	-	-	524	2.86[a]	$8.99\cdot10^{-4}$
**3c**		TMA-POSS	30	0.08	11	180.9[e]	227.6[e]<br>255.1[e]	250.4[e]<br>259.6[e]	378	14.6[c]	$8.44\cdot10^{-4}$
**3d**		TMA-POSS	40	0.11	8	182.1[e]	215.7[e]<br>256.3[e]	251[e]<br>259.2[e]	378	14.8[c]	$6.81\cdot10^{-4}$
**4**	[BMIm][TCM]					192.4[e]	-	-		0.36[b]	$8.4\cdot10^{-3}$
**4a**		AM0285	30	0.08	12	192.4[e]	-	-	547	1.94[a]	$2.8\cdot10^{-3}$
**5**	[BMIm][BETI]					190.7[e]	-	-		0.08[b]	$7.73\cdot10^{-4}$
**5a**		AM0285	30	0.16	5.3	191.6[e]	-	-	604	1.14[a]	$3.51\cdot10^{-4}$
**6**	[N_{2228}][[TFSI]					196.5[e]	-	-		0.04[b]	$2.07\cdot10^{-4}$
**6a**		AM0285	30	0.15	5.5	198.4[e]	-	-	569	0.93[a]	$1.54\cdot10^{-4}$
**6b**			5	0.021	45	196.5[e]	-	-	569	0.10[a]	$2.89\cdot10^{-4}$
**6c**			17	0.079	11.6	195.4[e]	-	-	569	0.15[a]	$2.24\cdot10^{-4}$
**6d**			40	0.22	3.5	195.9[e]	-	-	569	1.0[a]	$1.17\cdot10^{-4}$
**7**	[N_{1888}][[TFSI]					192.2[e]	-	-		0.04[b]	$2.08\cdot10^{-5}$
**7a**		AM0285	30	0.19	4	192.9[e]	-	-	578	0.82[a]	$1.60\cdot10^{-5}$
**8**	[$N_{122(2O1)}$][TFSI]					182.6[e]	-	-		0.1[b]	$7.88\cdot10^{-4}$
**8a**		AM0285	30	0.13	6.5	181.3[e]	-	-	568	1.00[a]	$5.68\cdot10^{-4}$

[a] Amount of water absorbed during sample preparation; [b]Amount of water determined for the purchased ILs by using KF titration; [c] Amount of water determined after preparation of TMA-POSS composites. Note that TMA-POSS nanoparticles contain 9wt% of water. [d] determined for hydrated material; [e] determined for anhydrous material

References

[1] Hipps, K. W.; Aplin, A. T. The Tricyanomethanide Ion: An Infrared, Raman, and Tunneling Spectroscopy Study Including Isotopic Substitution. J. Phys. Chem. 1985, 89 (25), 5459–5464.

[2] Dixon, D. A.; Calabrese, J. C.; Miller, J. S. Crystal and Molecular Structure of the Charge Transfer Salt of Decamethylferrocenium and Tricyanomethanide: [Fe(C5Me5)2]+•[C(CN)3]_. The Electronic Structure and Spectra of [C(CN)3]-. *J. Am. Chem. Soc.* 1986, *108* (10), 2582–2588.

[3] Weidinger, D.; Houchins, C.; Owrutsky, J. C. Vibrational Dynamics of Tricyanomethanide. *Chem. Phys. Lett.* 2012, *525–526*, 60–63.

[4] Romanos, G. E.; Zubeir, L. F.; Likodimos, V.; Falaras, P.; Kroon, M. C.; Iliev, B.; Adamova, G.; Schubert, T. J. S. Enhanced CO2 Capture in Binary Mixtures of 1-Alkyl-3- Methylimidazolium Tricyanomethanide Ionic Liquids with Water. *J. Phys. Chem. B* 2013, *117* (40), 12234–12251.

[5] Canongia Lopes, J. N. A.; Pádua, A. A. H. Nanostructural Organization in Ionic Liquids. *J. Phys. Chem. B* 2006, *110* (7), 3330–3335.

[6] Castner E.W. Jr., Margulis, C.J. Maroncelli, M. Wishart, J.F. Annu. Ionic liquids: structure and photochemical reactions, Rev. Phys. Chem. 62 (2011) 85.

[7] Holomb, R.; Martinelli, A.; Albinsson, I.; Lassègues, J. C.; Johansson, P.; Jacobsson, P. J.Ionic liquid structure: the conformational isomerism in 1-butyl-3-methyl-imidazolium tetrafluoroborate ([bmim][BF4]) Raman Spectrosc. 2008, 39, 793– 805

[8] Berg, R. W.; Deetlefs, M.; Seddon, K. R.; Shim, I.; Thompson, J. M. Raman and ab Initio Studies of Simple and Binary 1-Alkyl-3-methylimidazolium Ionic Liquids J. Phys. Chem. B 2005, 109, 19018–19025

[9] Castriota, M.; Caruso, T.; Agostino, R. G.; Cazzanelli, E.; Henderson, W. A.; Passerini, S. Raman Investigation of the Ionic Liquid N-Methyl-N-Propylpyrrolidinium Bis(Trifluoromethanesulfonyl)Imide and Its Mixture with LiN(SO 2CF3)2. *J. Phys. Chem. A* 2005, *109* (1), 92–96.

[10] Fukuda, S.; Takeuchi, M.; Fujii, K.; Kanzaki, R.; Takamuku, T.; Chiba, K.; Yamamoto, H.; Umebayashi, Y.; Ishiguro, S. ichi. Liquid Structure of N-Butyl-N-Methylpyrrolidinium Bis-(Trifluoromethanesulfonyl) Amide Ionic Liquid Studied by Large Angle X-Ray Scattering and Molecular Dynamics Simulations. *J. Mol. Liq.* 2008, *143* (1), 2–7.

[11] Ramenskaya, L. M.; Kudryakova, N. O.; Grishina, E. P. Conformation Features and Interaction of Pyrrolidinium-Based Ionic Liquids Immobilized with Silicon Dioxide: Infrared Spectroscopy. *J. Mol. Liq.* 2023, *382* (August 2022), 122025.

[12] Hatano, N.; Takekiyo, T.; Abe, H.; Yoshimura, Y. Effect of Counteranions on the Conformational Equilibrium of 1- Butyl- 3- methylimidazolium- Based Ionic Liquids. *Int. J. Spectrosc.* 2011, *2011* (1), 10–14.

[13] Rey, I.; Johansson, P.; Lindgren, J.; Lassegues, J. C.; Grondin, J.;Servant, L. Spectroscopic and Theoretical Study of (CF3SO2)2N- (TFSI-) and (CF3SO2)2NH (HTFSI) J. Phys. Chem. A 1998, 102, 3249

[14] Umebayashi, Y.; Mitsugi, T.; Fujii, K.; Seki, S.; Chiba, K.; Yamamoto, H.; Lopes, J. N. C.; Pádua, A. A. H.; Takeuchi, M.; Kanzaki, R.; Ishiguro, S. I. Raman Spectroscopic Study, Dft Calculations and Md Simulations on the Conformational Isomerism of n-Alkyl-n-Methylpyrrolidinium Bis-(Trifluoromethanesulfonyl) Amide Ionic Liquids. *J. Phys. Chem. B* 2009, *113* (13), 4338–4346.

[15] F.M. Vitucci, F. Trequattrini, O. Palumbo, J.-B. Brubach, P. Roy, A. Paolone, Infrared spectra of bis(trifluoromethanesulfonyl)imide based ionic liquids: Experiments and DFT simulations, Vibrational Spectroscopy, 74, 2014, 81-87

[16] Castriota, M.; Caruso, T.; Agostino, R. G.; Cazzanelli, E.; Henderson, W. A.; Passerini, S. Raman Investigation of the Ionic Liquid N-Methyl-N-Propylpyrrolidinium Bis(Trifluoromethanesulfonyl)Imide and Its Mixture with LiN(SO 2CF3)2. *J. Phys. Chem. A* 2005, *109* (1), 92–96
[17] Herstedt, M.; Smirnov, M.; Johansson, P.; Chami, M.; Grondin, J.; Servant, L.; Lassègues, J. C. Spectroscopic Characterization of the Conformational States of the Bis(Trifluoromethanesulfonyl)Imide Anion (TFSI-). *J. Raman Spectrosc.* 2005, *36* (8), 762–770.
[18] Brouillette, D.; Irish, D. E.; Taylor, N. J.; Perron, G.; Odziemkowski, M.; Desnoyers, J. E. Stable Solvates in Solution of Lithium Bis(Trifluoromethylsulfone)Imide in Glymes and Other Aprotic Solvents: Phase Diagrams, Crystallography and Raman Spectroscopy. *Phys. Chem. Chem. Phys.* 2002, *4* (24), 6063–6071.
[19] Loaiza, L. C.; Johansson, P. Li-Salt Doped Single-Ion Conducting Polymer Electrolytes for Lithium Battery Application. *Macromol. Chem. Phys.* 2022, *223* (8), 1–8.
[20] Paulechka, Y. U.; Kabo, G. J.; Blokhin, A. V.; Shaplov, A. S.; Lozinskaya, E. I.; Golovanov, D. G.; Lyssenko, K. A.; Korlyukov, A. A.; Vygodskii, Y. S. IR and X-Ray Study of Polymorphism in 1-Alkyl-3-Methylimidazolium Bis(Trifluoromethanesulfonyl)Imides. *J. Phys. Chem. B* 2009, *113* (28), 9538–9546.
[21] Paulechka, Y. U.; Kabo, G. J.; Emel'Yanenko, V. N. Structure, Conformations, Vibrations, and Ideal-Gas Properties of 1-Alkyl-3-Methylimidazolium Bis(Trifluoromethylsulfonyl)Imide Ionic Pairs and Constituent Ions. *J. Phys. Chem. B* 2008, *112* (49), 15708–15717.
[22] Lavan, S. N.; Ilic, S.; Viswanath, S.; Jain, A.; Assary, R. S.; Connell, J. G. Generalizable, Tunable Control of Divalent Cation Solvation Structure via Mixed Anion Contact Ion Pair Formation. *J. Mater. Chem. A* 2024, *12* (11), 6768–6776.
